# Reanalysis of the physical and mental health summary scores of dialysis versus conservative care in older patients with advanced chronic kidney disease: a critical appraisal

**DOI:** 10.1186/s13104-019-4765-3

**Published:** 2019-11-04

**Authors:** Wouter R. Verberne, Janneke Dijkers, Johannes C. Kelder, Wilbert T. Jellema, Johannes J. M. van Delden, Willem Jan W. Bos

**Affiliations:** 1 Department of Internal Medicine, St Antonius Hospital, Koekoekslaan 1, 3435 CM Nieuwegein, The Netherlands; 2 Department of Internal Medicine, Leiden University Medical Center, Leiden, The Netherlands; 3 Department of Clinical Epidemiology and Medical Statistics, St Antonius Hospital, Nieuwegein, The Netherlands; 4 Julius Centre for Health Sciences and Primary Care, University Medical Centre Utrecht, Utrecht, The Netherlands

**Keywords:** Health-related quality of life, Aged, Chronic kidney failure, Renal dialysis, Conservative treatment

## Abstract

**Objective:**

Non-dialytic conservative care is argued to be a reasonable treatment alternative for dialysis in selected older patients with advanced chronic kidney disease. We evaluated patient-relevant outcomes including health-related quality of life in a previous study. However, the scoring algorithm we used to calculate the physical and mental component summary scores of the Short Form-36 (SF-36) turned out to differ from comparable studies on this topic. The aim of this critical appraisal was to reanalyze the SF-36 summary scores in our patient cohort (≥ 70 years) using the more widely used scoring algorithm.

**Results:**

Patients on conservative care (*n* = 23) had lower physical and mental component summary scores compared to patients not yet started on dialysis (*n* = 39), but similar compared to patients on dialysis (*n* = 34). These findings were similar to our original findings and did not change the conclusions. Several scoring algorithms are used for the SF-36 summary scores. Researchers should be aware of this fact and should use the same scoring algorithm across similar studies in a specific field to increase comparability. Using the more widely used scoring algorithm, the recalculated SF-36 summary scores of our patient cohort can now be compared to other studies.

## Introduction

Non-dialytic conservative care is argued to be a reasonable treatment alternative for dialysis in selected older patients with advanced chronic kidney disease [1–3]. Comparative data on patient-relevant outcomes are, however, limited. Such data is needed to evaluate treatment effectiveness and may help to inform shared decision-making on preferred treatment [1, 4, 5]. We compared survival, health-related quality of life, and treatment burden in older patients choosing dialysis or conservative care in a previous observational cohort study [6]. The outcomes on health-related quality of life were assessed with the Kidney Disease Quality of Life Short Form (KDQOL-SF™) [7, 8], which includes the widely used Short-Form 36 Health Survey (SF-36, version 1) [9]. In the original publication [6], we reported the summary scores on physical health (physical component summary, PCS) and mental health (mental component summary, MCS) of the SF-36 as calculated with the scoring algorithm used by Kalantar-Zadeh et al. [10]. This scoring algorithm appeared to differ from the scoring algorithm used in similar studies reporting PCS and MCS scores in dialysis and conservative care patient groups [11–14]. Their scoring algorithm involved orthogonal rotation and norm-based scoring [15]. To enable meaningful comparisons of findings across studies, use of the same scoring algorithm would be preferable. The aim of this critical appraisal was (1) to recalculate the PCS and MCS scores in our patient cohort using the same scoring algorithm as in similar studies to enable comparisons of results across studies, and (2) to determine whether the recalculated PCS and MCS scores change our original study results or conclusions.

## Main text

### Methods

The methods of the original cohort study on dialysis versus conservative care were published elsewhere in detail [6]. To summarize, we retrospectively included patients aged ≥ 70 years old with advanced chronic kidney disease who chose dialysis or conservative care after shared decision-making in a non-academic teaching hospital in The Netherlands between October 31, 2004 and May 1, 2016. Health-related quality of life outcomes were assessed cross-sectionally in patients alive in 2015 and 2016 who consented to participate. We used the validated Dutch version of the KDQOL-SF™ to assess eight generic SF-36 domains and seven kidney disease-specific domains of health-related quality of life [7, 8]. Questionnaires were self-completed or interviewer-administered. The KDQOL-SF™ items were coded and scored according to the manual [16]. The eight SF-36 domains were used to calculate the PCS and MCS scores, by using the scoring algorithm by Kalantar-Zadeh et al. in our original publication [6, 10]. Scores range between 0 and 100; higher scores indicate better health-related quality of life. Baseline data, including age, sex, and comorbidities, were collected from electronic medical records. Comorbidities were scored according to the Davies comorbidity score, which is based on the presence of seven comorbid conditions and produces three risk groups (no comorbidity, intermediate comorbidity, severe comorbidity) [17]. All analyses were performed according to the original treatment choice. We compared the outcomes on health-related quality of life between patients on conservative care, patients who chose dialysis but were not yet treated with dialysis, and patients treated with dialysis. Students t-tests were used to test differences in the PCS and MCS scores between the three patient groups. Multiple linear regression analyses were performed on the PCS and MCS to evaluate their association with treatment pathway, adjusting for age, sex, Davies comorbidity score, and way of administration (self or by interviewer) with backward elimination. A *P* value < 0.05 was considered statistically significant. Statistical analyses were performed using IBM SPSS 24.0.

### New analysis

In this critical appraisal, we have recalculated the PCS and MCS scores by applying norm-based scoring with orthogonal rotation as used in similar studies reporting on this topic [11–15]. We used the SF-36 norms from the Dutch general population [18]. One-way analysis of variance (ANOVA) and Tukey HSD (Honestly Significant Difference) post hoc tests were used to test differences in the recalculated PCS and MCS scores respectively between and within the three patient groups. We checked the assumption of homogeneity. We have also repeated the multiple linear regression analyses on the recalculated PCS and MCS scores to evaluate their association with treatment modality (using dummy coding with conservative care as reference group), with adjustment for age, sex, Davies comorbidity score, and way of administration of the questionnaire (self or by interviewer).

## Results

99 of 128 eligible patients (77%) gave written informed consent to participate in the assessment of health-related quality of life outcomes. We excluded three patients from the analysis because of too many missing answers, resulting in 96 patients overall: 39 patients not yet started on dialysis, 34 patients started on dialysis, and 23 patients on conservative care. Compared to both patient groups on a dialysis pathway, patients on conservative care were older (mean age [standard deviation]: 83.8 [5.0] years in patients on conservative care, versus 79.8 [5.1] years in patients not yet started on dialysis, versus 80.1 [3.3] years in patients started with dialysis), and more often female (48% versus 31% versus 24%, respectively). There were no differences in Davies comorbidity score between the three patient groups (no comorbidity: 9% versus 13% versus 9%; intermediate comorbidity: 65% versus 59% versus 59%; severe comorbidity: 26% versus 28% versus 32%). Questionnaires were more frequently administered by an interviewer in patients started with dialysis (26% versus 18% versus 53%).

Table 1 shows the recalculated PCS and MCS scores, using the norm-based scoring algorithm with orthogonal rotation. There were significant differences between the three patient groups on the mean PCS as determined by one-way ANOVA (F(2,93) = 4.779, *P* = 0.01). Patients on conservative care scored lower on the PCS compared to patients not yet started on dialysis (*P* < 0.01), but similar compared to patients on dialysis (*P* = 0.38). A similar trend was seen in the unadjusted MCS scores, although no significant difference between the three patient groups was found (F(2,93) = 1.666, *P* = 0.20). Table 2 shows the multiple linear regression models on the recalculated PCS and MCS scores. These models confirmed the unadjusted findings on the PCS and MCS scores between the three patient groups: patients on conservative care scored lower on the PCS and MCS compared to patients not yet started on dialysis, but similar compared to patients on dialysis. An additional file shows the original results on the PCS and MCS scores based on the scoring algorithm by Kalantar-Zadeh et al. [10] (see Additional file 1: Tables S1 and S2).

**Table 1:** Physical and mental component summary scores using the norm-based scoring algorithm with orthogonal rotation

	Not yet started on dialysis (*n* = 39)	Started on dialysis (*n* = 34)	Conservative care (*n* = 23)	*P* value (Tukey HSD)
Physical component summary score, mean (SD)^a^	38.3 (10.4)	34.2 (9.3)	30.9 (7.2)	1: < 0.01^b^ 2: 0.38^c^ 3: 0.16^d^
Mental component summary score, mean (SD)^a^	52.8 (9.6)	50.5 (12.7)	47.5 (11.2)	1: 0.17 2: 0.58 3: 0.65

*SD* standard deviation; *Tukey HSD*, Tukey’s Honestly Significant Difference post hoc test

^a^Scores range between 0 and 100; higher scores indicate better health-related quality of life

^b^Not yet started on dialysis versus Conservative care

^c^Started on dialysis versus Conservative care

^d^Not yet started on dialysis *versus* Started on dialysis

**Table 2:** Multiple linear regression models of the physical and mental component summary scores, using the norm-based scoring algorithm with orthogonal rotation, in patients choosing dialysis but not yet started on dialysis (*n* = 39), in patients started on dialysis (*n* = 34), and in patients choosing conservative care (*n* = 23)

A—Physical component summary score^a^	B	95% CI for B	Beta	*P* value
Constant	33.17	28.93 to 37.42		
Female vs. male	− 4.85	− 8.89 to − 0.81	− 0.24	0.02
Treatment pathway (conservative care as reference)				
Not yet started on dialysis vs. conservative care	6.61	1.79 to 11.43	0.34	< 0.01
Started on dialysis vs. conservative care	2.20	− 2.79 to 7.20	0.11	0.38

B—Mental component summary score^b^	B	95% CI for B	Beta	*P* value
Constant	43.97	39.88 to 48.06		
Interviewer-administration vs. self-administration	13.44	9.07 to 17.80	0.56	< 0.001
Treatment pathway (conservative care as reference)				
Not yet started on dialysis vs. conservative care	6.45	1.48 to 11.41	0.28	0.01
Started on dialysis vs. conservative care	− 0.58	− 5.80 to 4.64	− 0.03	0.83

*CI* confidence interval, vs. versus

^a^Physical Component Summary score model: R^2^ = 0.15, F(3,92) = 5.24, *P *= 0.002. Results were similar when additionally adjusted for age, Davies comorbidity score, and way of administration

^b^Mental component summary score model: R^2^ = 0.31, F(3,92) = 14.02, *P *< 0.001. Results were similar when additionally adjusted for age, sex, and Davies comorbidity score

## Discussion

In this critical appraisal, we recalculated the summary scores on physical health (PCS) and mental health (MCS) of the SF-36 in our cohort of patients ≥ 70 years old with advanced chronic kidney disease who chose either dialysis or conservative care, using the same scoring algorithm as in similar studies. Patients who had chosen dialysis but were not yet started on dialysis had higher scores on the recalculated PCS and MCS compared to patients on conservative care. No differences were observed in the recalculated PCS and MCS scores between patients on dialysis or conservative care. These findings were similar to our original findings on the PCS and MCS [6]. We therefore conclude that the reanalysis of the PCS and MCS did not change the direction or significance of the results, interpretations, or conclusions of our original article. Conservative care could be a viable treatment option in selected older patients with advanced chronic kidney disease to achieve similar health-related quality of life outcomes compared to dialysis.

The main advantage of the recalculated PCS and MCS is that it enables appropriate comparison of our results to other studies using the same scoring algorithm. This is specifically relevant when comparing the absolute scores on the PCS and MCS across studies, as only the absolute values have changed in our reanalysis. We found that the recalculated PCS and MCS scores in our patient groups were within the range of results of comparable studies [11–14]. The mean or median PCS score in patients on a dialysis pathway, including patients not yet started on dialysis and patients on dialysis, was between 25 and 38 in previous studies (34.2 and 38.3 in our patient groups on a dialysis pathway), and 18–34 in patients on conservative care (30.9 in our patient group on conservative care) [11–14]. Similarly for the MCS, studies found a mean or median MCS score of 43–50 in patients on a dialysis pathway (50.5 and 52.8 in our patient groups on a dialysis pathway), and 46–52 in patients on conservative care (47.5 in our patient group on conservative care) [11–14]. When comparing the PCS and MCS scores between patients on a dialysis pathway and patients on conservative care, previous studies found no statistically differences between the treatment pathways. We only observed a higher PCS score in patients not yet started on dialysis compared to the conservative care patient group, but this finding might be explained by the age difference between the patient groups. To conclude, our results on physical and mental health, including the absolute PCS and MCS scores, were consistent with the findings in comparable studies.

After publishing our original study, we have discovered that at least four different scoring algorithms have been used to calculate the PCS and MCS of the SF-36 [10, 15, 19, 20]. The most used scoring algorithms appear to be norm-based scoring with orthogonal rotation or oblique rotation [15, 19]. While it is unclear which scoring algorithm would be best to use [20–26], an important advantage of norm-based scoring is that such scores are standardized relative to the general population scores which makes interpretation easier. We think it is an important warning to other researchers that different scoring algorithms are being used to calculate the PCS and MCS of the SF-36. We recommend that the scoring algorithm used should be reported explicitly in publications, including which norm scores were used in case of norm-based scoring. Moreover, we recommend to use the same scoring algorithm in similar studies in a specific field to enable comparisons of results across studies, and allow qualitative synthesis of study findings such as in a meta-analysis. Such standardized approach increases the efficacy of studies and patient input.

## Limitations

Our reanalysed findings on physical and mental health in older patients on dialysis versus conservative care had several limitations similar to those described in the original publication [6]. Treatment allocation bias and confounding were potential flaws due to the observational study design and non-random treatment decision. We observed that patients who chose conservative care were older and more often female than patients who chose dialysis, while comorbidity level was similar between patient groups. We adjusted for several confounders in the multivariable regression analysis to overcome this problem. Yet, residual confounding might be possible. Other limitations to our findings were the small sample size and cross-sectional assessment of health-related quality of life. Large comparative studies with longitudinal assessment of health-related quality of life outcomes in older patients on dialysis and conservative care are needed to confirm current findings.

## Supplementary information

**Additional file 1.** Includes two supplementary tables that show our original results on the PCS and MCS scores [6], based on the scoring algorithm by Kalantar-Zadeh et al. [10]. **Table S1** shows the original mean PCS and MCS scores in the three patient groups. **Table S2** shows the original multiple linear regression models on the PCS and MCS.

## Data Availability

The datasets used and/or analysed during the current study are available from the corresponding author on reasonable request.

## Abbreviations

CI: confidence interval
KDQOL-SF™: Kidney Disease Quality of Life Short Form
MCS: mental component summary
PCS: physical component summary
SF-36: Short Form-36
SPSS: Statistical Package for the Social Science
Vs.: versus

## References

[1] Davison SN, Levin A, Moss AH, Jha V, Brown EA, Brennan F (2015). Executive summary of the KDIGO controversies conference on supportive care in chronic kidney disease: developing a roadmap to improving quality care. Kidney Int..

[2] Farrington K, Covic A, Aucella F, Clyne N, de Vos L, Findlay A (2016). Clinical Practice Guideline on management of older patients with chronic kidney disease stage 3b or higher (eGFR < 45 mL/min/173 m2). Nephrol Dial Transplant..

[3] Williams AW, Dwyer AC, Eddy AA, Fink JC, Jaber BL, Linas SL (2012). Critical and honest conversations: the evidence behind the “Choosing Wisely” campaign recommendations by the American Society of Nephrology. Clin J Am Soc Nephrol.

[4] Evangelidis N, Tong A, Manns B, Hemmelgarn B, Wheeler DC, Tugwell P (2017). Developing a Set of Core Outcomes for Trials in Hemodialysis: an International Delphi Survey. Am J Kidney Dis.

[5] Verberne WR, Das-Gupta Z, Allegretti AS, Bart HAJ, van Biesen W, García-García G (2019). Development of an international standard set of value-based outcome measures for patients with chronic kidney disease: a report of the international consortium for health outcomes measurement (ICHOM) CKD Working Group. Am J Kidney Dis.

[6] Verberne WR, Dijkers J, Kelder JC, Geers ABM, Jellema WT, Vincent HH (2018). Value-based evaluation of dialysis versus conservative care in older patients with advanced chronic kidney disease: a cohort study. BMC Nephrol..

[7] Hays RD, Kallich JD, Mapes DL, Coons SJ, Carter WB (1994). Development of the kidney disease quality of life (KDQOL) instrument. Qual Life Res.

[8] Korevaar JC, Merkus MP, Jansen MA, Dekker FW, Boeschoten EW, Krediet RT (2002). Validation of the KDQOL-SF: a dialysis-targeted health measure. Qual Life Res.

[9] The RAND-36 Health Survey. https://www.rand.org/health/surveys_tools/mos/36-item-short-form.html.

[10] Kalantar-Zadeh K, Kopple JD, Block G, Humphreys MH (2001). Association among SF36 quality of life measures and nutrition, hospitalization, and mortality in hemodialysis. J Am Soc Nephrol.

[11] Brown MA, Collett GK, Josland EA, Foote C, Li Q, Brennan FP (2015). CKD in elderly patients managed without dialysis: survival, symptoms, and quality of life. Clin J Am Soc Nephrol.

[12] Da Silva-Gane M, Wellsted D, Greenshields H, Norton S, Chandna SM, Farrington K (2012). Quality of life and survival in patients with advanced kidney failure managed conservatively or by dialysis. Clin J Am Soc Nephrol.

[13] Seow YY, Cheung YB, Qu LM, Yee AC (2013). Trajectory of quality of life for poor prognosis stage 5D chronic kidney disease with and without dialysis. Am J Nephrol.

[14] Iyasere O, Brown EA, Johansson L, Davenport A, Farrington K, Maxwell AP (2019). Quality of life with conservative care compared with assisted peritoneal dialysis and haemodialysis. Clin Kidney J..

[15] Ware J, Kosinski M, Keller S (1994). SF-36 physical and mental health summary scales: a user’s manual.

[16] Hays R, Kallich J, Mapes D, Coons S, Amin N, Carter W, et al. Kidney Disease Quality of Life Short Form (KDQOL-SF™), Version 1.3: A Manual for Use and Scoring. Santa Monica, CA: RAND,1995.

[17] Davies SJ, Russell L, Bryan J, Phillips L, Russell GI (1995). Comorbidity, urea kinetics, and appetite in continuous ambulatory peritoneal dialysis patients: their interrelationship and prediction of survival. Am J Kidney Dis.

[18] Aaronson NK, Muller M, Cohen PD, Essink-Bot ML, Fekkes M, Sanderman R (1998). Translation, validation, and norming of the Dutch language version of the SF-36 Health Survey in community and chronic disease populations. J Clin Epidemiol.

[19] Hays R, Prince-Embury S, Chen H (1998). R-36 HSI: RAND-36 Health Status Inventory.

[20] Farivar SS, Cunningham WE, Hays RD (2007). Correlated physical and mental health summary scores for the SF-36 and SF-12 Health Survey. VI. Health Qual Life Outcomes..

[21] Taft C, Karlsson J, Sullivan M (2001). Do SF-36 summary component scores accurately summarize subscale scores?. Qual Life Res.

[22] Ware JE, Kosinski M (2001). Interpreting SF-36 summary health measures: a response. Qual Life Res..

[23] Cunningham WE, Nakazono TT, Tsai KL, Hays RD (2003). Do differences in methods for constructing SF-36 physical and mental health summary measures change their associations with chronic medical conditions and utilization?. Qual Life Res.

[24] Ware JE, Gandek B, Kosinski M, Aaronson NK, Apolone G, Brazier J (1998). The equivalence of SF-36 summary health scores estimated using standard and country-specific algorithms in 10 countries: results from the IQOLA Project.International Quality of Life Assessment. J Clin Epidemiol.

[25] Simon GE, Revicki DA, Grothaus L, Vonkorff M (1998). SF-36 summary scores: are physical and mental health truly distinct?. Med Care.

[26] Hays RD, Morales LS (2001). The RAND-36 measure of health-related quality of life. Ann Med.

