# Supplementary material for: Reanalysis of the physical and mental health summary scores of dialysis versus conservative care in older patients with advanced chronic kidney disease: a critical appraisal
Source: BMC Res Notes. 2019 Nov 4;12:722. doi: 10.1186/s13104-019-4765-3 (PMC6829838; doi:10.1186/s13104-019-4765-3)
Supplement: Supplementary file 1 — Additional file 1. Includes two supplementary tables that show our original results on the PCS and MCS scores [6], based on the scoring algorithm by Kalantar-Zadeh et al. [10]. Table S1 shows the original mean PCS and MCS scores in the three patient groups. Table S2 shows the original multiple linear regression models on the PCS and MCS. [file 13104_2019_4765_MOESM1_ESM.docx]

**Additional file 1**

**Additional Table S1.** Original results on the physical and mental component summary scores [6], based on the scoring algorithm by Kalantar-Zadeh et al [10]

|  | Not yet started on dialysis  (*n* = 39) | Started on dialysis  (*n* = 34) | Conservative care  (*n* = 23) | *P* value  (Tukey HSD)^b^ |
| --- | --- | --- | --- | --- |
| Physical Component Summary score, mean (SD)^a^ | 56.0  (20.6) | 48.1  (20.9) | 40.2  (16.2) | 1: <0.01^c^  2: 0.31^d^  3: 0.20^e^ |
| Mental Component Summary score, mean (SD)^a^ | 68.5  (17.2) | 62.0  (22.0) | 54.2  (19.7) | 1: 0.02  2: 0.31  3: 0.34 |

*SD*, standard deviation; *Tukey HSD*, Tukey’s Honestly Significant Difference post hoc test.

^a^= Scores range between 0 and 100; higher scores indicate better health-related quality of life

^b^= There was an overall difference between the three patient groups on the mean PCS score as determined by one-way ANOVA (F(2,93) = 4.754, *P* = 0.01), and on the mean MCS score (F(2,92) = 3.923, *P* = 0.02).

^c^= Not yet started on dialysis *versus* Conservative care

^d^= Started on dialysis *versus* Conservative care

^e^= Not yet started on dialysis *versus* Started on dialysis

**Additional Table S2.** Original multiple linear regression models of the PCS and MCS [6], based on the scoring algorithm by Kalantar-Zadeh et al [10], in patients choosing dialysis but not yet started on dialysis (*n* = 39), in patients started on dialysis (*n* = 34), and in patients choosing conservative care (*n* = 23)

|  | B | 95% CI for B | Beta | *P* value |
| --- | --- | --- | --- | --- |
| Physical Component Summary score^a^ |  |  |  |  |
| Constant | 41.31 | 32.54 to 50.09 |  |  |
| Female *vs.* Male | -10.01 | -18.28 to -1.73 | -0.23 | 0.02 |
| Interviewer-administration *vs.* Self-administration | 14.23 | 5.63 to 22.84 | 0.33 | 0.001 |
| Treatment pathway (Conservative care as reference) |  |  |  |  |
| Not yet started on dialysis *vs.* Conservative care | 15.24 | 5.46 to 25.03 | 0.37 | 0.003 |
| Started on dialysis *vs.* Conservative care | 1.58 | -8.87 to 12.04 | 0.04 | 0.76 |
| Mental Component Summary score^b^ |  |  |  |  |
| Constant | 48.82 | 41.30 to 56.34 |  |  |
| Interviewer-administration *vs.* Self-administration | 20.49 | 12.41 to 28.57 | 0.48 | < 0.001 |
| Treatment pathway (Conservative care as reference) |  |  |  |  |
| Not yet started on dialysis *vs.* Conservative care | 16.03 | 6.90 to 25.16 | 0.39 | 0.001 |
| Started on dialysis *vs.* Conservative care | 2.01 | -7.67 to 11.69 | 0.05 | 0.68 |

*CI,* confidence interval; *vs.,* versus.

^a^= Physical Component Summary score model: R^2^= 0.22, F(4,91) = 6.36, *P <* 0.001. Results were similar when additionally adjusted for age and Davies comorbidity score

^b^= Mental Component Summary score model: R^2^= 0.28, F(3,91) = 11.77, *P* < 0.001. Results were similar when additionally adjusted for age, sex, and Davies comorbidity score

**References**

6. Verberne WR, Dijkers J, Kelder JC, Geers ABM, Jellema WT, Vincent HH, et al. Value-based evaluation of dialysis versus conservative care in older patients with advanced chronic kidney disease: a cohort study. BMC Nephrol. 2018;19(1):205.

10. Kalantar-Zadeh K, Kopple JD, Block G, Humphreys MH. Association among SF36 quality of life measures and nutrition, hospitalization, and mortality in hemodialysis. J Am Soc Nephrol. 2001;12(12):2797-806.
